# Immune disturbance leads to pulmonary embolism in COVID-19 more than classical risk factors: a clinical and histological study

**DOI:** 10.1007/s11739-023-03383-9

**Published:** 2023-08-17

**Authors:** Sebastiano Cicco, Antonio Vacca, Federica Albanese, Nicola Susca, Vanessa Desantis, Arianna Magistro, Gerardo Cazzato, Gerolamo Cicco, Sara Sablone, Christel Cariddi, Marialuisa Sveva Marozzi, Cristiana Catena, Gabriele Brosolo, Stefano Marcante, Giuseppe Ingravallo, Lidia Dalfino, Gianfranco Lauletta, Fabrizio Pappagallo, Antonio Giovanni Solimando, Salvatore Grasso, Eugenio Maiorano, Francesco Introna, Leonardo Alberto Sechi, Roberto Ria

**Affiliations:** 1 COVID Section, Unit of Internal Medicine “Guido Baccelli”, Department of Precision and Regenerative Medicine and Ionian Area–(DiMePRe-J), U.O.C. Medicina Interna “Guido Baccelli”, University of Bari Aldo Moro, Policlinico di Bari, Piazza Giulio Cesare 11, 70124 Bari, Italy; 2 Division of Internal Medicine, Department of Medicine, University of Udine, Building 8, 33100 Udine (UD), Italy; 3 Department of Precision and Regenerative Medicine and Ionian Area–(DiMePRe-J), Pharmacology Section, University of Bari Aldo Moro Medical School, Bari, Italy; 4 Department of Precision and Regenerative Medicine and Ionian Area–(DiMePRe-J), Section of Pathology, University of Bari Aldo Moro, 70124 Bari, Italy; 5 Department of Medical and Surgical Sciences for Children and Adults, University of Modena and Reggio Emilia, 41121 Modena, Italy; 6 Department of Interdisciplinary Medicine, Section of Forensic Medicine, University of Bari Aldo Moro, Piazza Giulio Cesare, 11, 70124 Bari, Italy; 7 Department of Precision and Regenerative Medicine and Ionian Area–(DiMePRe-J), Section of Anesthesiology and Intensive Care, Ospedale Policlinico, University of Bari Aldo Moro, Piazza G. Cesare 11, 70124 Bari, Italy

**Keywords:** Immune cells, Pulmonary embolism, SARS-CoV-2, NETosis, HMGB1, Embolism risk factor

## Abstract

COVID-19 induces endotheliitis and one of the main complications is enhanced coagulation. The incidence of pulmonary embolism (PE) in COVID-19 (CPE) has increased and clinical features for a rigorous analysis still need to be determined. Thus, we evaluated the clinical characteristics in CPE and the immune infiltration that occurred. Between January 1 and December 31, 2021, 38 patients were affected by CPE (9 ICU, 19 males/19 females, 70.18 ± 11.24 years) out of 459 COVID-19 cases. Controls were subjects who were evaluated for PE between January 1 2015, and December 31, 2019 (92 patients, 9 ICU, 48 males/45 females, 69.55 ± 16.59 years). All patients underwent complete physical examination, pulmonary computed tomography, laboratory tests, D-dimer, and blood gas analysis. There were no differences in laboratory tests or D-dimer. In patients with CPE, pO2, alveolar–arterial oxygen difference (A-aDO2), oxygen saturation %, and the ratio between arterial partial pressure of oxygen (PaO2) and fraction of inspired oxygen (FiO2), P/F, were significantly increased. There were no differences in PaCO2. Platelet count was inversely correlated to P/F (*r* = − 0.389, *p* = 0.02) but directly to A-aDO2 (*r* = 0.699, *p* = 0.001) only in patients with CPE. Histology of lung biopsies (7 CPE/7 controls) of patients with CPE showed an increase in CD15^+^ cells, HMGB1, and extracellular MPO as a marker of NETosis, while no significant differences were found in CD3^+^, CD4^+^, CD8^+^, and intracellular MPO. Overall, data suggest that CPE has a different clinical setting. Reduced oxygen content and saturation described in Patients with CPE should not be considered a trustworthy sign of disease. Increased A-aDO2 may indicate that CPE involves the smallest vessels as compared to classical PE. The significant difference in NETosis may suggest the mechanism related to thrombi formation.

**Supplementary Information:**

The online version contains supplementary material available at 10.1007/s11739-023-03383-9.

## Introduction

In 2020, new pandemic challenge was sustained by SARS-CoV-2 infection causing coronavirus disease 2019 (COVID-19). A massive systemic inflammation is a typical disease feature due to the infection and consequent immune response characterized by an unrestrainable cytokine storm [1]. A broad spectrum of clinical manifestations has been described during the SARS-CoV-2 infection [2]. However, cardiovascular and pulmonary involvement is a prominent disease feature associated with a worsened prognosis [3, 4]. Cardiovascular involvement is one of the leading factors for clinical worsening [5]. On the other hand, endothelial dysfunction, diffuse vascular inflammation, hypercoagulability, and immune thrombosis expose patients to a significant risk of arterial and venous thromboembolism (VTE) and pulmonary embolism (PE) [6].

Abnormal coagulation parameters, such as prothrombin time, activated partial thromboplastin time, fibrin degradation products, and D-dimer values, imply activation of the COVID-19-related coagulation and sequelae [7] and increased D-dimer values result to be poor prognostic indicators [8]. Clinical characteristics for a rigorous analysis still need to be determined and hence data on the difference between pulmonary embolisms in patients with COVID-19 (CPE) and those in non-COVID-19 (PE) are needed. Due to the difference in risk factors between CPE and PE, many authors suggest an immune-mediated thrombosis associated with COVID-19 [9]. However, few data were reported on molecular mechanisms involved in pulmonary thrombosis differentiating CPE from PE. Authors relate the immune activation to possible endothelial damage leading to an increased clot formation via von Willebrand factor and immune-mediated cytokines release [10, 11].

This study evaluated oxygenation, cardiac, pulmonary, and laboratory characteristics in CPE compared to PE in patients admitted into the Internal Medicine Department and the Intensive Care Unit. Due to our hypothesis on possible pathophysiological processes [11], we also evaluated inflammatory lung tissue infiltration and immune profiling in post-mortem analysis to understand possible differences between CPE and PE.

## Methods

### Patients

This observational retrospective study was conducted in conformity with the Good Clinical Practice Guidelines of the Italian Ministry of Health and the ethical guidelines of the Declaration of Helsinki (as revised and amended in 2004) following the approval by the Ethics Committee of the University of Bari Medical School (Code number 6645/2020).

We evaluated 459 patients with COVID-19 admitted to the Internal Medicine Department from January 1 2020 to December 31, 2021. We found 38 patients (8.28%) affected by CPE (19 males/19 females, aged 70.18 ± 11.24 years). Among these, we excluded patients affected by Left Ventricular Failure (4 patients) and those with incomplete echocardiograms (5 patients). Thus, we selected 29 patients with CPE (14 males/15 females, aged 70.86 ± 11.83 years) to be included in the study. We also selected patients admitted directly from Emergency Room to the Intensive Care Unit for COVID-19 and PE. Out of 162 patients, we selected 9 (5.55%) (5 males/4 females, aged 68.00 ± 9.38 years) who presented the same inclusion/exclusion criteria described above. All of them were hypoxemic; all were treated with azithromycin after admission. All patients with COVID-19 were affected by interstitial pneumonia, mild-to-moderate between Internal Medicine patients, and severe between ICU patients. As controls, data from 92 patients with PE (48 males/45 females, aged 69.55 ± 16.59 years) evaluated between January 1, 2015 and December 31, 2019 were examined. We selected 84 patients admitted into the Internal Medicine Department (44 males and 40 females, aged 70.88 ± 16.44 years). We also included 9 patients (4 males/5 females, aged 69.00 ± 11.42 years) SARS-CoV-2-negative PE who were admitted directly from Emergency Room to ICU at the same time as our cohort. At diagnosis, complete physical examination was performed in both groups of patients; patients also underwent computerized pulmonary tomography, laboratory tests, D-dimer, and blood gas analysis (BGA). In addition, all patients were evaluated according to PaO2/FiO2 (P/F) ratio, which indicates the severity of respiratory failure because of affected lung parenchyma, lung functionality, and response to treatment. We calculated the National Early Warning Score (NEWS) including heart rate, respiratory rate, body temperature, systolic blood pressure, Glasgow Coma Scale (GCS), oxygen saturation percentage, Sequential Organ Failure Assessment (SOFA) score, and oxygen was administered to have a comprehensive and comparable clinical evaluation.

Both CPE and control patients with PE showed good hemodynamic status; thus, low molecular weight heparin was administered, as indicated in 2019 European Society of Cardiology (ESC) guidelines for PE [12]. We used enoxaparin 100 U/kg twice a day for PLT count of more than 50,000 cells/mm^3^ and glomerular filtration rate > 30 ml/min. Patients with CPE also received steroids (Dexamethasone for ten days 6 mg i.v. once a day, up to 8 mg i.v. once a day for severe ICU patients). Oxygen up to non-invasive ventilation support was given to those with P/F < 150 to support respiration at best and reducing respiratory effort.

At patients’ admission, we measured levels of erythrocyte sedimentation rate (ESR), creatinine, uric acid, C-reactive protein (CRP), and blood count using commercial laboratory diagnostic kits.

### Ultrasound evaluation

Due to the different and unstandardized method, we chose to include ultrasound evaluation only in patients admitted in the Internal Medicine Department. Ultrasound evaluation was performed with a standardized method by the same expert operator, including a transthoracic echocardiogram with Doppler evaluation (ETG) [13]. A 2.5-MHz probe in the left lateral decubitus position was used to perform all ultrasound evaluations. All patients were screened for deep vein thrombosis (DVT) at admission using compressive ultrasound with a 12-MHz linear probe on the lower limbs in the supine position.

Aortic root diameter (Ao) was measured in parasternal long-axis view during both systolic (AoS) and diastolic phases (AoD) by an ECG-guided point measurement. In the same position, end-diastolic measurements of interventricular septum thickness (IVS), posterior wall thickness (PWD), and left ventricular internal diameter (LVD) were obtained using one-dimensional echocardiography (M-mode). According to international guidelines [14], we used the Devereux formula for M-mode diameters to calculate the left ventricular mass (LVM). Relative wall thickness (RWT) was calculated using the internationally validated formula [14]. Tricuspid annular plane systolic excursion (TAPSE) and right atrial area (RA) were measured in an apical four-chamber view (A4C). A4C view was also applied to measure left ventricular end-diastolic volume (LVEDV), ejection fraction (EF), and left atrial volume (LAV). Ao, LAV, and LVM were indexed to body surface area (BSA) and height 2.7 (LVMi2.7). We used a subcostal view (SC) to measure inferior vena cava diameter (CVD). We obtained Doppler measurements in an A4C B-mode view. The tricuspid regurgitation velocity (TRV) was determined using a continuous wave Doppler curve of the tricuspid regurgitation (TR) trace. According to the simplified Bernoulli equation (*p* = 4[TRmax]2), we chose the peak value of TRV to measure the pressure difference between the right ventricle and right atrium (RA). RA filling pressure was estimated from the diameter and respirophasic variability of the inferior vena cava during normal breathing. We applied a derived sum of RA filling and TR pressures to estimate pulmonary arterial pressure (PAP). In the same view, transmitral velocity E and A and septal velocity e’ were calculated with pulsed-wave Doppler in tissue Doppler imaging (TDI) mode. A combination of mitral ratio E/A and E/e’ was applied to assess and stratify diastolic dysfunction.

### Histological analysis

Seven patients with CPE and seven controls underwent a post-mortem evaluation to test our hypothesis about NETosis and high mobility group box 1 (HMGB1) interaction in CPE [11]. Patients were comparable in age, sex, comorbidities, and clinical evolution. Lung samples were analyzed to evaluate immune cell infiltration. Considering the viral nature of the disease, we analyzed the T cell lymphocyte and neutrophil infiltration to assess if cell-mediated inflammation has a role in neutrophil response and NETosis. Samples were fixed in neutral 10% buffered formalin, dehydrated, and enclosed in paraffin. 5 µm thick slices were taken from the paraffin-embedded blocks, deparaffinized, rehydrated, and routinely stained with Hematoxylin–Eosin (H&E). Antibodies for the following markers were used to perform immunohistochemistry: polyclonal rabbit anti-human CD3 (Agilent, DAKO Omnis, Cat.GA503, 1:50 dilution), monoclonal mouse anti-human CD4 (Agilent, DAKO Omnis, Carpinteria,CA,USA, Cat.M7310, 1:50 dilution), monoclonal mouse anti-human CD8 (Novacastra Laboratories Ltd., Cat. NCL-L-CD8-4B11, 1:50 dilution), and monoclonal mouse anti-human CD15 (Agilent, DAKO Omnis, Cat. GA062, 1:500 dilution). Myeloperoxidase (MPO) was evaluated to assess neutrophils extracellular traps (NETs) with polyclonal rabbit anti-human myeloperoxidase (Agilent, DAKO Omnis, Cat. GA511, 1:500 dilution). We used SARS-CoV-2 Spike Protein S1 Monoclonal Antibody (HL6), (ThermoFisher Scientific, Cat. MA5-36,247, 1:250 dilution) to evaluate SARS-CoV-2 positivity in lung samples,

The expression of HMGB1 evaluated with polyclonal rabbit anti-HMGB1 serum (Ab18256, Abcam, Cambridge, USA) was used to study immune cell infiltration. We pre-treated blocks on PT-LINK (DAKO) instrument with EDTA [EnVision Flex, Target Retrieval Solution, High Ph (50×), DAKO] for CD3, CD4, CD8, CD15 antibodies and Citrate [EnVision Flex, Target Retrieval Solution, Low Ph (50×), DAKO] for the HMGB1 antibody. The density of CD4 + and CD8 + cells was measured with immunohistochemistry in 10 fields at 400 × magnification. One field measured 140 μm in length and 110 μm in width, and the total amplitude was 15,400 μm squared. We used a Reichert Polyvar 2 microscope with a JTV digital camera and a Trinitron monitor (Sony). HMGB1 expression was assessed highlighting the chromogen signal on the samples of plasma membrane, nucleus, cytoplasm, or extracellular medium. The relative expression level was calculated by adding the degree of staining intensity (grade 0 = no staining; grade 1 = weak staining; grade 2 = moderate staining; grade 3 = intense staining) with the percentage of mass extension (score 0: < 1%; score 1: 1–25%; score 2: 26–50%; value 3: 51–74%; score 4: ≥ 75%). The resulting final scores were rated as high (if > 3) or low (if ≤ 3). After processing, two expert pathologists scored the samples as previously standardized [13]. The final value reported represents the mean of the two values. Similarly, we analyzed the chromogen signal of MPO both intracellularly and in the extracellular space.

### Statistics

Data were analyzed using GraphPad Prism software (La Jolla, CA, USA) and expressed as means ± S.D. for parametric data and median and interquartile range [IQR]. The distribution of dichotomous values was analyzed with chi-square test. Regarding non-normally distributed data, we performed a non-parametric Mann–Whitney test for comparisons and Spearman distribution for correlations. Normally distributed data were studied with parametric unpaired *t*-test for comparisons and Pearson distribution. Statistical significance was indicated with a value of *p* < 0.05. We performed a Cox regression after testing proportional Hazard in the Schoenfeld residuals test to understand if different factors were associated with mortality. The survival and Hazard Ratio was evaluated using the Log-Rank (Mantel–Cox) test and displayed using **Kaplan–Meier** graphs.

## Results

### Clinical and laboratory results

The CPE and the controls groups overlapped in age, sex distribution, body mass index (BMI), and body surface area (BSA) (Table 1). Comorbidities were significantly fewer in CPE (*p* = 0.0005) (Table 1), while controls presented a higher incidence of malignancies (*p* = 0.001), and DVT (*p* = 0.0005). Similar results were found in non-ICU controls who were more likely to present arterial hypertension and heart disease (*p* = 0.005 and *p* = 0.041, respectively). ICU patients presented a similar higher rate of comorbidities among controls, but no statistical significance was found, maybe due to the small cohort.

**Table 1:** Clinical characteristics of the patients studied

	All population			ICU patients			Internal medicine patients		
	CPE	Controls	*p* value	CPE	Controls	*p* value	CPE	Controls	*p* value
Age	70.18 ± 11.24	69.55 ± 16.59	0.831	68.00 ± 9.38	69.00 ± 11.42	0.842	70.86 ± 11.83	70.88 ± 16.44	0.667
Sex (M/F)#	19/19	44/48	0.848	5/4	4/5	1.000	14/15	40/44	1.000
Weight (kg)	77.62 ± 8.76	76.74 ± 18.93	0.875	85.34 ± 12.24	79.86 ± 8.78	0.475	77.62 ± 8.76	75.94 ± 18.84	0.875
Height (cm)	1.67 ± 0.09	1.68 ± 0.06	0.708	1.68 ± 0.10	1.67 ± 0.08	0.693	1.68 ± 0.06	1.67 ± 0.09	0.756
BSA (m^2^)	1.86 ± 0.16	1.85 ± 0.23	0.516	2.11 ± 0.36	1.93 ± 0.25	0.503	1.87 ± 0.13	1.85 ± 0.23	0.864
BMI (kg/m^2^)	28.63 ± 4.54	27.21 ± 6.01	0.486	31.57 ± 5.06	29.00 ± 4.73	0.345	27.42 ± 2.96	27.16 ± 6.02	0.767
Comorbidities@	1 [0–2]	2 [1–3]	0.0005	1 [1, 2]	3 [1.5–3]	0.006	1 [0–2]	2 [1–3]	** < 0.0001**
Arterial Hypertension (%)#	16 (42.10)	56 (60.87)	0.081	7 (77.78)	4 (44.44)	0.335	9 (31.03)	52 (62.65)	**0.005**
Heart disease (%)#	8 (21.05)	35 (38.04)	0.100	3 (33.33)	3 (33.33)	1.000	5 (17.24)	32 (38.55)	**0.041**
Atrial Fibrillation (%)#	3 (7.89)	13 (14.13)	0.394	1 (11.11)	2 (22.22.)	1.000	2 (6.89)	11 (13.25)	0.509
Active malignancies (%)#	5 (13.16)	40 (43.48)	**0.001**	0 (0.00)	2 (22.22)	0.476	5 (17.24)	38 (45.78)	**0.008**
Diabetes (%)#	7 (18.42)	24 (26.09)	0.498	1 (11.11)	3 (33.33)	0.576	6 (20.69)	21 (25.30)	0.802
Immune mediated disease (%)#	4 (10.53)	16 (17.39)	0.427	1 (11.11)	3 (33.33)	0.576	3 (10.34)	13 (15.66)	0.758
Deep vein thrombosis (%)#	4(10.53)	38 (41.30)	**0.0005**	0 (0.00)	3 (33.33)	0.206	4 (13.79)	35 (42.17)	**0.006**
WELLS-PE score@	0 [0–3]	5.5 [3–7]	** < 0.0001**	3 [0–4-125]	3.5 [1.5–5.625]	**0.050**	0 [0–1.375]	5.5 [3–7.5]	** < 0.0001**
CHA2DS2VASc@	2 [1–3]	4 [3–6]	** < 0.0001**	3.5 [1.25–4]	3.5 [1.5–5.25]	0.659	2 [1–3]	4.5 [3–6]	** < 0.0001**
HAS-BLED@	1 [0–1]	2 [1–3]	** < 0.0001**	0.5 [0–1.75]	1 [0–1.75]	0.588	1 [0–1]	2 [1–3]	** < 0.0001**
PESI@	81.5 [76.75–112.5]	110 [84–130.8]	**0.009**	94 [71–121.8]	134 [117.5–181.5]	**0.0005**	80 [76.25–99.25]	106 [82.50–130]	**0.019**
GCS@	15 [15–15]	15 [15–15]	0.194	15 [15–15]	15 [5.5–15]	**0.019**	15 [15–15]	15 [15–15]	0.438
SOFA@	3 [1.5–5]	1 [0–6]	0.510	3.5 [2–4]	1 [0–6]	0.701	3 [1–5]	3 [1.25–6.25]	0.875
NEWS@	3 [2–5]	8 [4.5–10]	**0.009**	6 [3.5–9]	8 [4.5–10]	0.456	2 [2, 3]	2 [1–3]	0.567
SBP (mmHg)	128.32 ± 16.91	125.15 ± 19.56	0.392	124.00 ± 20.52	118.71 ± 20.90	0.475	129.50 ± 15.99	126.58 ± 20.55	0.348
DBP (mmHg)	77.29 ± 12.13	74.00 ± 11.93	0.161	74.78 ± 15.09	67.86 ± 15.20	0.195	78.07 ± 11.15	73.84 ± 11.97	**0.025**
HR (bpm)	84.97 ± 20.81	90.32 ± 21.03	0.196	95.50 ± 37.92	110.86 ± 25.28	0.246	82.07 ± 12.43	88.71 ± 19.33	**0.018**
Temperature (°C)	36.36 ± 0.81	36.23 ± 0.79	0.615	36.34 ± 0.75	37.67 ± 1.69	**0.019**	36.64 ± 0.52	36.46 ± 0.94	0.432

Values are shown as mean ± SD or median [IQR] for non-parameters variables

The significant *p* values are in bold

*BMI* = Body mass index, *BSA* = body surface area, *DBP* diastolic blood pressure, *GCS* Glasgow coma scale, *HR* heart rate, *NEWS* national early warning score, *PESI* pulmonary embolism severity Index, *SBP* systolic blood pressure

*p* values are from *t*-test for parametric values, Mann–Whitney for non-parametric (@), or Chi-squared test (#) for distribution

Using the WELL-PE scoring system on the coagulative milieu or disease severity, controls were found more at risk of pulmonary embolism (*p* < 0.0001). Also, controls presented a higher risk of blood clots formation evaluated by the CHA_2_D_2_-VASc scoring system (*p* < 0.0001) and bleeding evaluated by the HAS-BLED system (*p* < 0.0001) (Table 1). These results were similar in Internal medicine patients, while ICU ones had an increased WELL-PE score (*p* = 0.05) but similar CHA_2_D_2_-VASc and HAS-BLED. Finally, an increased pulmonary embolism severity index (PESI) was found in controls among all patients (*p* = 0.009) as well as among ICU (*p* = 0.0005) and non-ICU ones (*p* = 0.019) (Table 1).

CPE and PE control patients did not differ in the Glasgow Coma Scale (GCS), National Early Warning Score (NEWS) and Sequential Organ Failure Assessment (SOFA) (Table 1). CPE presented a higher diastolic blood pressure (DBP) (*p* = 0.025) and heart rate (*p* = 0.018) (Table 1). The two groups did not differ in the length of hospitalization and death rate (Table 1).

There were no differences in D-dimer (supplementary table 1). Patients with CPE presented an increased ESR (*p* = 0.047) but a decreased aPTT (*p* = 0.037) and uric acid (*p* = 0.01). The same results were found among Internal medicine patients, while no difference emerged among ICU ones (Supplementary table 1). At the same time, controls presented a higher incidence of heart damage evaluated using NT-proBNP (*p* = 0.034 all populations, *p* = 0.017 in ICU patients, *p* = 0.004 in Internal medicine patients) (supplementary table 1). On the contrary, Troponin I was significantly higher among non-ICU controls compared to patients with CPE (*p* = 0.044), but no difference was found among ICU patients (Supplementary table 1). No other difference was found in laboratory tests between the groups.

### Respiratory data

All patients received supplemental oxygen therapy, and there was no difference in the type of oxygen supply assigned at admission (Table 2). The CPE group showed an increase in PaO2 compared to controls (83.62 ± 35.13 vs. 77.33 ± 32.78 mmHg). However, it resulted significant only among Internal medicine patients (*p* = 0.03) (supplementary table 2). Compared to controls, patients with CPE showed a higher difference of oxygen concentration between alveoli and arteries (A-aDO2; *p* = 0.0002 all populations, *p* = 0.049 in ICU patients, *p* = 0.0002 in Internal medicine patients). Similarly, the oxygen saturation percentage results lower in patients with CPE contrasted with all population (*p* = 0.048) and ICU (*p* = 0.053) but results higher among Internal medicine patients (*p* = 0.02) compared to control group. Similarly, the P/F was decreased in CPE (*p* = 0.001), especially among Internal medicine patients (*p* = 0.008), while through ICU patients, it results not significant (supplementary table 2).

**Table 2:** Characteristics of hospitalization between the two groups

	CPE	Controls	*p* value
Oxygen supply at admission	38 (100%)	92 (100%)	1.000
Non-invasive ventilation	3	7	1.000
High-flow nasal cannula	4	4	0.237
Venturi mask	22	63	0.238
Endotracheal intubation	9	9	0.060
Length of stay (days)	16.5 [8–25]	9 [6–15]	**0.011**
Dead patients (number at 60 days) (%)	10 (26.32)	28 (30.43)	0.536
Dead patients (number at 60 days) excluding ICU (%)	6 (20.69 on 29 pts)	20 (24.09 on 83 pts)	0.803
Death during hospitalization (number) (%)	7 (18.42)	15 (16.30)	0.803
Death during hospitalization (number) excluding ICU (%)	2 (6.89 on 29 pts)	10 (11.90 on 83 pts)	0.728

The significant *p* values are in bold

Patients with CPE presented a PLT count inversely correlated with P/F (*r* = − 0.425, *p* = 0.01) but directly correlated with A-aDO2 (*r* = 0.679, *p* = 0.001) (supplementary Fig. 1A). No similar findings were found among controls (supplementary Fig. 1B).

### Data on echocardiography

Left heart walls were thicker in patients with CPE compared to controls. Interventricular septum and posterior wall diameters were increased among patients with CPE (*p* = 0.026 and *p* = 0.040, respectively) (Table 3). However, patients with CPE presented a not significantly decreased interior LV diameter and LV end-diastolic volume. Thus, the comparable mass between the two groups assessed with the Devereux formula may be related to these findings (Table 3).

**Table 3:** Heart ultrasound parameters in the two groups of patients hospitalized in internal medicine ward; values are shown as mean ± SD or median for non-parameters variables

	CPE	Controls	*p *value
Left heart			
Interventricular septum (IVS) (mm)	13.36 ± 2.42	11.86 ± 1.56	**0.026**
LV diameter (LVedD) (mm)	43.91 ± 5.68	47.46 ± 6.03	0.101
Posterior wall diameter (PWD) (mm)	13.25 ± 2.24	12.00 ± 1.52	**0.040**
RWT	0.51 ± 0.12	0.51 ± 0.08	0.959
LVM (gr)	208.9 ± 51.54	218.4 ± 62.92	0.672
LVMi (gr/m^2^)	109.9 ± 29.33	113.7 ± 36.19	0.768
LVMih (gr/m^2.7^)	51.02 ± 15.43	53.35 ± 15.02	0.681
LV volume (LVedV) (ml)	92.40 ± 29.12	97.96 ± 41.19	0.698
LAV (ml)	60.18 ± 20.32	61.30 ± 28.93	0.908
Lavi (ml/m^2^)	31.50 ± 10.48	33.30 ± 15.97	0.744
LAVih (gr/m^2.7^)	13.38 ± 6.69	15.13 ± 7.46	0.506
AoD (mm)	32.27 ± 3.26	31.52 ± 3.42	0.537
AoDi (mm/m^2^)	17.26 ± 1.70	17.26 ± 2.30	0.999
EF (%)	62.00 ± 3.85	58.14 ± 10.95	0.121
Mitral velocity E_m_ (cm/s)	59.55 ± 14.80	59.58 ± 23.55	0.997
Mitral velocity A_m_ (cm/s)	71.50 ± 14.13	70.42 ± 26.03	0.902
E/A_m_	0.87 ± 0.36	0.92 ± 0.44	0.746
Mitral velocity e’_m_ (cm/s)	8.30 ± 3.05	6.77 ± 2.39	**0.028**
E/e’_m_	8.30 ± 3.04	9.90 ± 6.22	0.275
Diastolic dysfunction #			
None	9	16	0.749
Grade 1	14	54	
Grade 2–3	6	13	
Right heart			
TRV (m/s)	2.03 ± 0.64	2.28 ± 0.79	0.398
PAPs (mmHg)	25.60 ± 10.16	32.64 ± 15.33	0.063
Inferior cava vein diameter (mm)	14.90 ± 6.20	16.36 ± 4.46	0.429
Right ventricle area (RVA) (cm^2^)	17.88 ± 4.77	20.11 ± 5.00	0.117
Right ventricle basal diameter (RVd1) (mm)	37.67 ± 3.67	36.71 ± 7.82	0.774
Right ventricle outflow tract (RVOT) (mm)	27.00 ± 4.97	28.67 ± 7.09	0.727
Right ventricle area (RVA) (cm^2^)	21.67 ± 6.21	22.76 ± 6.12	0.650
Pulmonary acceleration time (Act) (msec)	101.0 ± 14.31	80.75 ± 38.14	0.134
Pulmonary artery diameter (Pd) (mm)	20.50 ± 4.66	22.80 ± 2.59	0.375
TAPSE (mm)	25.44 ± 3.94	23.46 ± 6.02	0.197
TAPSE/PAPs (mm/mmHg)	0.99 ± 0.85	1.12 ± 0.71	0.543

The significant *p* values are in bold

*p* values are from *t*-test and Chi-squared test (#)

Patients with CPE showed a Left Ventricle (LV) with regular isochoric diastolic relaxation pattern evaluated by e’ velocity, while it decreased in control ones (Table 3). Thus, e’ velocity of LV significantly decreased among control patients (*p* = 0.028) (Table 3).

A significant trend (*p* = 0.063) in increased Pulmonary Artery Pressure (PAPs) was found among patients with CPE when compared to controls, but no other significant differences were found. Similarly, no difference was found in right ventricular-arterial uncoupling evaluated as the ratio between Tricuspid Annular Plane Systolic Excursion (TAPSE) and PAPs.

### Survival and outcomes

**Fig. 1:**
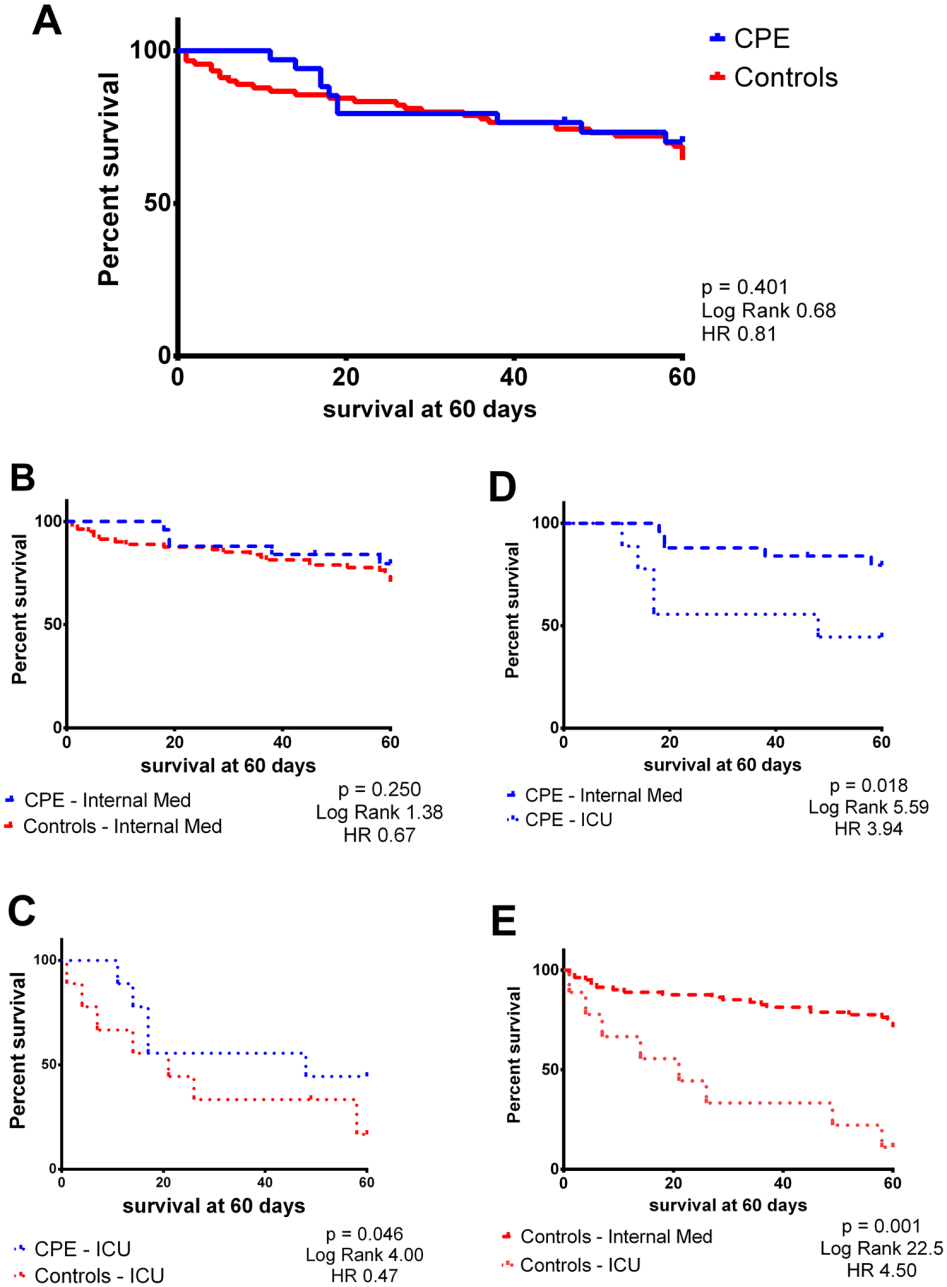
Survival comparison between the groups, comparing CPE and controls in all populations (panel **A**), patients admitted to non-ICU ward (panel **B**) and those who were admitted to ICU (panel **C**). The remaining panels show the comparison between ICU and internal medicine survival in CPE (panel **D**) and Controls (panel **E**)

There was no difference in cardiovascular and oncological comorbidities, as well as rate of intubation and related pneumonia. Patients with CPE presented a significant increase in length of hospitalization (CPE 16.5 [8–25] vs Controls 9 [6–15] days, *p* = 0.011). On the contrary, there was an overlapping length of hospitalization between the two groups (CPE 10 vs Controls 9 days) among Internal medicine patients. There was also no difference in in-hospital mortality and death rate (Table 2) and a similar survival (Fig. 1A) especially if ICU patients were excluded (Fig. 1B). However, patients with CPE who were admitted to ICU presented an increased survival compared to similar control patients (Patients with CPE 48 months vs controls 21 months, *p* = 0.046) (Fig. 1C), although admission in ICU is a risk for fatal evolution in both CPE and control patients (HR 3.94 and 4.50 respectively) (Fig. 1D, E). There was no difference in determinants of death between the two groups considering both clinical and echocardiographic parameters in Cox regression analysis (Supplementary Tables 3 and 4).

### Histological examination of lung specimens

The study aimed to investigate the connection between the presence of immune cells and the occurrence of blood clots among patients with CPE. In this perspective, we examined the lung tissues of both groups of patients in post-mortem analysis in order to determine the extent of vascular damage.

**Fig. 2:**
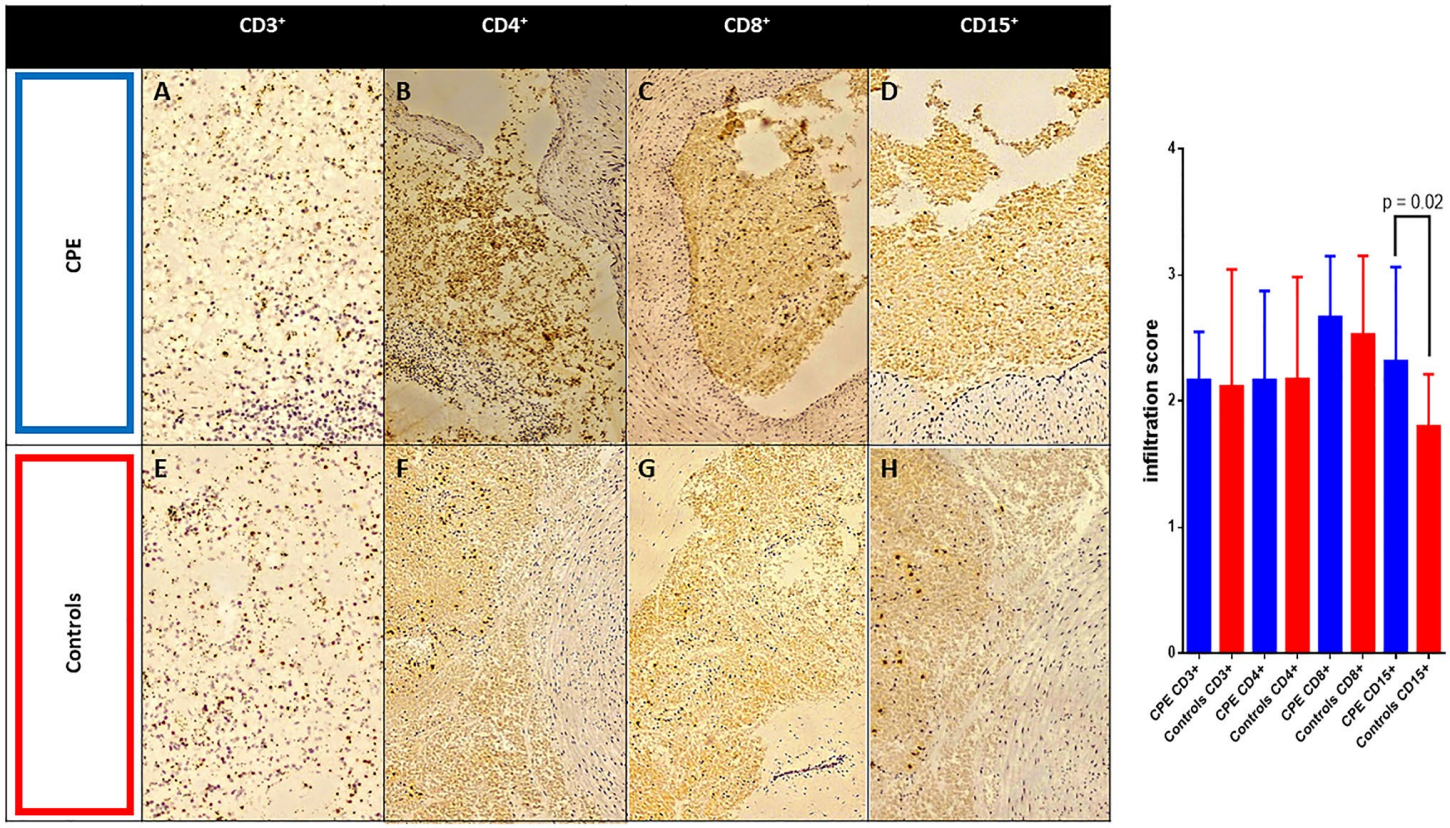
Immune cell infiltration (CD3^+^, CD4^+^, CD8^+^ and CD15^+^) in biopsy samples from lung tissue of COVID-19 pulmonary embolism (CPE) patients (**A**, **B**, **C**, **D**) and controls (**E**, **F**, **G**, **H**). Representative immuno-histochemistry (original magnification: 10×) is shown in the top and middle panels. Histograms shows quantification of infiltration via pathological score. Results are given as mean ± S.D. of three independent experiments for each field. The bar in panel **A** indicates the length of 100 µm and stands for all panel in the figure

**Fig. 3:**
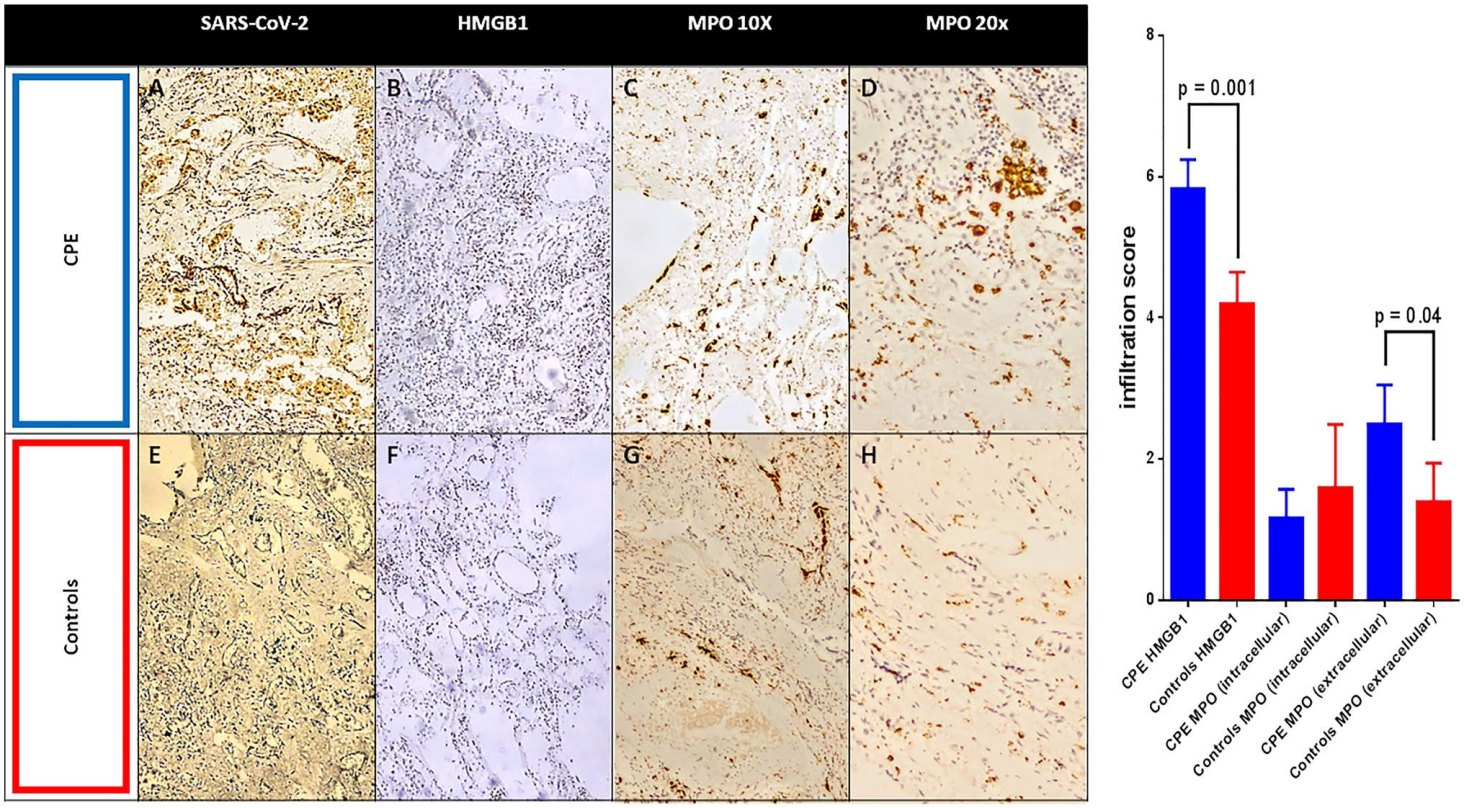
Panel **A** and **E** show the search of SARS-CoV-2 virus in the same samples. Expression level of inflammation-associated cytokines of vascular injury HMGB1 and neutrophil extracellular traps (NETs) marker myeloperoxidase (MPO) in biopsy samples from lung tissue of COVID-19 pulmonary embolism (CPE) patients (**B**, **C**) and controls (**F**, **G**). Representative immuno-histochemistry (original magnification: 10×) is shown in the top and middle panels. MPO expression was shown also in 20 × magnification (panel **D** for CPE and panel **H** for controls). Histograms shows quantification of infiltration via pathological score. Results are given as mean ± SD of three independent experiments for each field. The bar in panel **A** indicates the length of 100 µm similar for all 10 × images (**A**, **B**, **C**, **E**, **F**, **G**), while the bar in panel **D** stands for 200 µm in 20 × magnification (panel **D** and **H**)

There was no difference in CD3 + , CD4 + , and CD8 + cell infiltration among patients with CPE compared to control ones (Fig. 2). To evaluate neutrophilic vessel wall infiltration, CD15 + immune cells were stained, and a significant expression in CPE-derived vessels than in controls was found (*p* = 0.019) (Fig. 2). HMGB1 staining, which labels immunological inflammatory cells, was also significantly higher in patients with CPE (Fig. 3). MPO analysis revealed a decrease in the intracellular compound during a dramatic increase in the extracellular one in patients with CPE (Fig. 3).

## Discussion

Thromboembolic complications, including venous thromboembolism (VTE), are a typical clinical feature of patients with COVID-19 [15]. Pulmonary Embolism (PE) may be an actual life-threatening condition [12]. Many parameters (such as uric acid, aPTT) or specific scores were considered helpful as prognostic values of PE, as demonstrated by evidence [16, 17]. Our patients with CPE presented a shortened aPTT associated with an increased risk of VTE [18]. Similarly, uric acid was decreased in COVID-19-related PE compared to controls. Patients with CPE also presented fewer comorbidities than controls and lower WELLS-PE and PESI scores values. All these findings suggest an overall lower risk of pulmonary embolism and systemic coagulation in patients with CPE compared to control group. This suggests that CPE may cause localized thrombi formation more than a systemic activation. Hence, the prognosis was more favorable.

COVID-19-related coagulopathy is also demonstrated by the elevation of D-dimer associated with a systemic state of thrombosis, representing the effect of crosstalk between the innate immune system and coagulation [19]. This mechanism results from vascular inflammation, endothelial dysfunction, and hypercoagulability related to SARS-CoV-2 infection [20]. Immune cell infiltration and cytokines release [11] lead to consequent inflammation and induce the activation of a coagulative cascade [19]. The increase of ESR found in Internal medicine patients suggests the role of systemic inflammation in developing a pro-thrombotic state with thromboembolic complications in this setting. In addition, the decreased NLR and the lymphocytes in ICU patients suggest the role of cellular immunity as first factor. However, histological data on immune infiltration in patients with CPE demonstrated the presence of neutrophilic vessel wall infiltration, evidenced by significantly higher levels of CD15 + immune cells in CPE-derived vessels.

Moreover, MPO analysis revealed a vivid NET activation compared to controls, which play an essential role in host defense and acute inflammation [21]. Finally, patients with CPE showed an HMGB1 increase, which labels immunological inflammatory cells. CD15 + cells may undergo necrosis during CPE, thus shedding NETs that contribute to the HMGB1 increase. Also, histologic results confirmed the significant infiltration by the immune system, representing a key feature of chemotactic cytokines release and vascular damage. These results indicate a considerable neutrophil extracellular trap (NET) activation in patients with CPE. All data suggest an association with inflammation-related vascular damage in patients with CPE. CD15 + cells may undergo necrosis during COVID-19 CPE, thus shedding NETs that contribute to the HMGB1 increase. In line with the previous observations, histology results support the idea that immune cell infiltration is a crucial feature of vascular damage and chemotactic cytokines release. COVID-19-related inflammation with immune dysregulation, endothelial dysfunction, and consequent vascular damage could be the basis of the insurgence of pulmonary embolism. In addition, HMGB1 and CD15 + may be considered differential markers expressed in patients with CPE. However, further bench analysis is needed to establish the exact mechanism.

Notably, endothelial dysfunction as an activator of thrombosis [19] could result from the tropism of SARS-CoV-2 for the endothelium [22, 23]. Vascular endothelium results as one of the main targets of the virus [22] due to its entry properties via angiotensin-converting enzyme 2 (ACE2). Increasing evidence suggests that SARS-CoV-2 directly targets endothelial cells, promoting the release of proinflammatory and prothrombotic molecules [24]; thus, the altered endothelium homeostasis may lead to widespread endotheliitis.

COVID-19-related endothelial dysfunction in our patients with CPE may be prompted by higher diastolic blood pressure (DBP) compared to controls suggesting the correlation between a higher DBP and endotheliitis [25]. Vascular remodeling during vasculitis increases vascular stiffness but a lower DBP [13]. Therefore, a possible role of endothelial dysfunction as a lack of vascular modulation may be considered. However, other studies are necessary to investigate the correlation between DBP and COVID-19-related endotheliitis.

A large study has demonstrated that COVID-19-related PE is primarily described in smaller vessels than non-COVID-PE [26], and a meta-analysis demonstrated that patients with COVID-19 were more affected by pulmonary embolisms unrelated to DVT [27]. This may explain the differences that we found in echocardiographic patterns. Overall, data suggest no differences in pulmonary embolism-related heart damage considering the underlying disease. The negligible increase in PAPs in controls reflects the different comorbidities and disease severity. This should be explained by patients with CPE’ excellent correct heart performance as shown by a suitable mean value of right heart-pulmonary artery coupling. Since COVID-19 affects the lung heavily, evidences described a reduced right heart function compared to non-patients with COVID-19 [22, 28–30], especially in case of severe disease [28]. Therefore, we evaluated the proper heart parameters to understand whether an echocardiographic difference may be helpful in the diagnostic workout. However, our results did not indicate a difference between COVID-19 and non-patients with COVID-19 when pulmonary embolism was detected.

Both ICU and Internal medicine patients presented a more severe P/F although both CPE and controls patients were in oxygen treatment. This may be part of COVID-19-related “happy hypoxemia” already described in these patients [31] although it represents a better condition compared to classical PE. In fact, patients with CPE may benefit from steroids to reduce inflammation and ventilation to support the respiratory system that remains perfused as lower PAPs showed. On the contrary, classical PE presented a mismatch in ventilation–perfusion coupling that may not be easily overcome due to mechanical obstruction of blood flow not related to local inflammation.

Patients with CPE showed that the PLT value increase correlates to two respiratory parameters. The increase in PLTs mainly results from a systemic inflammatory state, and PLTs are important coordinators of inflammation and immune response [32]. Other studies showed that PLTs promote an inflammatory hypercoagulable phenotype associated with induced and amplified endotheliopathy in COVID-19 [33]. PLT count presented an inverse correlation to P/F but a direct correlation to A-aDO2. In patients with CPE, P/F as a parameter of lung dysfunction may heavily depend on the PLT count. Another study demonstrates that an antiplatelet therapy might improve the ventilation/perfusion ratio in Patients with COVID-19 by preventing and interfering with forming clots in lung capillary vessels and modulating megakaryocytes’ function and PLTs adhesion [34]. Thus, decreasing PLT values may be associated with improving P/F ratio and respiratory function.

On the other hand, A-aDO2 can be used as another parameter of lung dysfunction [35]. An abnormally increased A-aDO2 suggests a defect in diffusion and V/Q mismatch. In our patients with CPE, an increase of A-aDO2 directly correlates with increased PLT count. The direct correlation of PLTs to the increase in alveolar–capillary interface and the inverse correlation to P/F ratio may all be part of the exact inflammatory mechanism. Moreover, the association between PLT count and P/F and A-aDO2 parameters indicates a crucial role of PLTs in COVID-19-related inflammation and the importance of antiplatelet therapy to improve patients’ respiratory outcomes.

Furthermore, the finding of results so similar in patients admitted to ICU stressed once again the idea that COVID-19 may play a key role in PE despite its association to a better outcome.

There are some limitations to this study. First, a few enrolled patients were affected by PE despite two centers being uncounted. We need more data on more severe patients who underwent intensive care at the very beginning. Second, controls were patients evaluated before the COVID-19 pandemic. This choice reduces a possible confounding factor of occult infections. However, the different temporal distributions must be encountered in interpreting the result.

Similarly, data on oxygenation may be considered according to the respiratory treatment the patients received in first aid admission. On the contrary, we can give a good picture of low-intensity care patients affected by PE during COVID-19. Similarly, our data support the observation that patients with COVID-19 presented an immune-mediated thrombosis of lung medium and small vessels. Moreover, we found that patients affected by pulmonary embolism in COVID-19 are more likely to recover due to fewer comorbidities. Finally, according to blood gas analysis, our patients received oxygen treatment to correct their respiratory failure. Thus, P/F was evaluated without a standardized FiO_2_, and respiratory values may be considered cautiously. However, our data picture a real-life setting of CPE care.

In conclusion, COVID-PE appears to have a different clinical course. Reduced oxygenation described in PE may not be considered a sign of disease. The increased A-aDO2 may indicate that COVID-PE involved the smallest vessels compared to classical PE. Different inflammatory characterization relates to the different observations we found. Intensive Care admission results the main risk factor for a fatal outcome, especially in non-COVID patients.

### Supplementary Information

Below is the link to the electronic supplementary material.

Supplementary file1 (JPG 99 KB)

Supplementary file2 (DOCX 23 KB)

## Abbreviations

A4: Four-chamber view
A-aDO2: Alveolo-arterial difference of oxygen
Ao: Aortic root diameter
AoD: Aortic root diameter during the diastolic phase
AoS: Aortic root diameter during the systolic phase
aPTT: Activated partial thromboplastin time
BGA: Blood gas analysis
BMI: Body mass index
CPE: Patients with COVID-19 pulmonary embolism
CRP: C-reactive protein
CVD: Vena cava diameter
DVT: Deep vein thrombosis
EF: Ejection fraction
ESC: European society of cardiology
ESR: Erythrocyte sedimentation rate
GCS: Glasgow coma scale
H&E: Hematoxylin–eosin
HDL: High-density lipoprotein
HMGB1: High mobility group box 1
IQR: Interquartile range
IVS: Interventricular septum
LAV: Left atrial volume
LDL: Low-density lipoprotein
LV: Left ventricle
LVD: Left ventricular internal diameter
LVEDV: Left ventricular end-diastolic volume
LVM: Left ventricular mass
LVMi2.7: LVM indexed to height 2.7
MPO: Myeloperoxidase
NETs: Neutrophils extracellular traps
NEWS: National early warning score
NT-proBNP: N-terminal prohormone of brain natriuretic peptide
PAP: Pulmonary artery pressure
PE: Patients with non-COVID-19 pulmonary embolism
P/F: Ratio between arterial partial pressure of oxygen and fraction of inspired oxygen
PESI: Pulmonary embolism severity index
PLT: Platelet
PWD: Posterior wall thickness
RA: Right atrium
RWT: Relative wall thickness
SC: Sub-costal
SOFA: Sequential organ failure assessment
TAPSE: Tricuspid annular plane systolic excursion
TDI: Tissue Doppler imaging
TRV: Tricuspid regurgitation velocity
TR: Tricuspid regurgitation
VTE: Venous thromboembolism
