# Supplementary figures and images for: Immune disturbance leads to pulmonary embolism in COVID-19 more than classical risk factors: a clinical and histological study
Source: Intern Emerg Med. 2023 Aug 17;18(7):1981–93. doi: 10.1007/s11739-023-03383-9 (PMC10543807; doi:10.1007/s11739-023-03383-9)

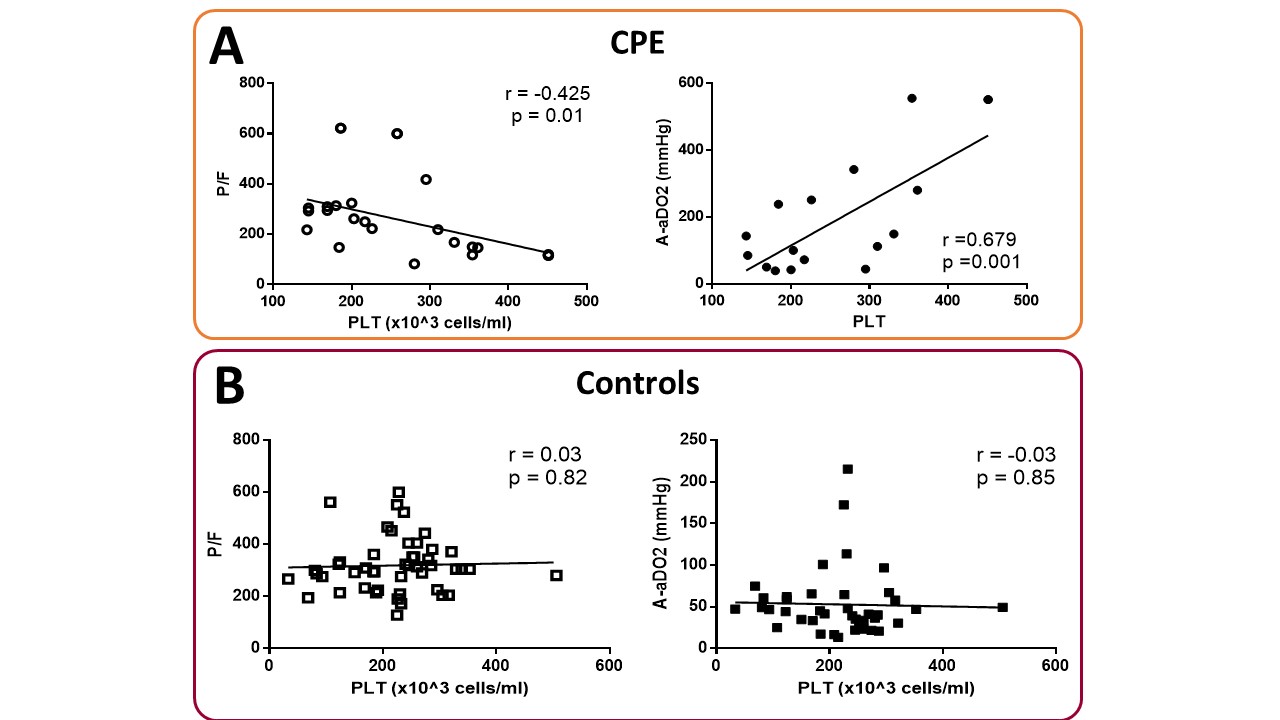

Supplement: Supplementary file 1 — Supplementary file1 (JPG 99 KB) [file 11739_2023_3383_MOESM1_ESM.jpg]
