# Supplementary material for: Immune disturbance leads to pulmonary embolism in COVID-19 more than classical risk factors: a clinical and histological study
Source: Intern Emerg Med. 2023 Aug 17;18(7):1981–93. doi: 10.1007/s11739-023-03383-9 (PMC10543807; doi:10.1007/s11739-023-03383-9)
Supplement: Supplementary file 2 — Supplementary file2 (DOCX 23 KB) [file 11739_2023_3383_MOESM2_ESM.docx]

**Supplementary Table 1:** Laboratory findings. Values are shown as mean ± SD or median for non-parameters variables. P values are from t-test.

|  | Total population | | | ICU patients | | | Internal medicine patients | | |
| --- | --- | --- | --- | --- | --- | --- | --- | --- | --- |
|  | CPE | Controls | P value | CPE | Controls | P value | CPE | Controls | P value |
| Hb (gr/dl) | 12.90 ±1.90 | 11.67 ± 1.98 | **0.001** | 13.63 ± 2.26 | 10.76 ± 1.60 | **0.007** | 12.40 ±1.55 | 11.72 ± 2.04 | **0.033** |
| WBC (x10^3 cells/ml) | 9.34 ± 4.35 | 9.83 ± 4.93 | 0.594 | 9.18 ± 4.27 | 13.28 ± 6.28 | 0.124 | 9.74 ± 3.29 | 9.14 ± 4.90 | 0.950 |
| PLT (cells/ml) | 221.92 ± 82.09 | 245.19 ± 125.87 | 0.297 | 195.55 ± 64.19 | 254.0 ± 123.78 | 0.227 | 249.6 ± 85.9 | 242.1 ± 138.0 | 0.581 |
| N% | 79.38 ± 12.69 | 74.95 ± 14.05 | 0.096 | 85.08 ± 9.89 | 91.03 ± 3.25 | 0.106 | 79.99 ± 11.92 | 73.31 ± 13.82 | 0.131 |
| L% | 14.18 ± 9.45 | 16.93 ± 10.37 | 0.162 | 9.60 ± 6.05 | 4.99 ± 2.35 | **0.049** | 13.96 ± 8.85 | 18.08 ± 9.77 | 0.223 |
| NLR | 9.79 ± 9.09 | 18.63 ± 35.42 | 0.081 | 12.37 ± 7.05 | 22.51 ± 11.73 | **0.041** | 8.99 ± 9.60 | 7.09 ± 7.78 | 0.286 |
| PLR | 258.44 ± 183.41 | 305.97 ± 376.77 | 0.305 | 354.48 ± 304.58 | 1020 ± 1885 | 0.311 | 250.5 ± 131.3 | 219.5 ± 148.1 | 0.286 |
| C-reactive protein | 153.83 ± 53.62 | 161.48 ± 79.34 | 0.718 | 132.94 ± 84.15 | 186.64 ± 109.27 | 0.271 | 91.15 ± 69.39 | 68.99 ± 73.77 | 0.982 |
| Erythrocyte Sedimentation rate (mm1h) | 58.00 ± 24.31 | 44.57 ± 29.24 | **0.047** |  |  |  | 58.00 ± 24.31 | 44.57 ± 29.24 | **0.047** |
| eGFR (ml/min) | 74.95 ± 23.15 | 73.07 ± 30.02 | 0.704 | 70.55 ± 25.46 | 57.78 ± 36.69 | 0.403 | 71.40 ± 29.31 | 76.31 ± 22.70 | 0.802 |
| Uric Acid (mg/dl) | 3.88 ± 2.14 | 5.59 ± 2.10 | **0.010** |  |  |  | 3.88 ± 2.14 | 5.59 ± 2.10 | **0.010** |
| LDH | 387.14 ± 190.88 | 347.96 ± 270.75 | 0.487 | 514.43 ± 239.44 | 826.00 ± 542.85 | 0.282 | 344.7 ± 156.3 | 302.1 ± 208.4 | 0.491 |
| PT-INR | 1.18 ± 0.32 | 1.18 ± 0.23 | 0.985 | 1.31 ± 0.45 | 1.36 ± 0.29 | 0.788 | 1.14 ± 0.26 | 1.15 ± 0.23 | 0.687 |
| aPTT | 1.04 ± 0.14 | 1.15 ± 0.31 | **0.037** | 1.07 ± 0.15 | 1.36 ± 0.79 | 0.305 | 1.03 ± 0.14 | 1.15 ± 0.21 | **0.004** |
| Fibrinogen | 400.79 ± 129.34 | 378.77 ± 164.35 | 0.485 | 417.67 ± 172.09 | 520.00 ± 326.50 | 0.418 | 394.7 ± 114.0 | 375.3 ± 127.6 | 0.261 |
| Troponin I (pg/mL) | 70.16 ± 207.99 | 134.15 ± 334.30 | 0.176 | 23.78 ± 36.44 | 51.19 ± 72.93 | 0.475 | 37.04 ± 69.68 | 155.0 ± 364.0 | **0.044** |
| NT-proBNP (pg/mL) | 745.20 ± 1052 | 3201.90 ± 5308.14 | **0.034** | 1105 ± 1683 | 5346.37± 4137.27 | **0.017** | 601.4 ± 684.8 | 3373.2 ± 5959.1 | **0.004** |
| D-dimer | 19655.18 ± 59160.25 | 8208.18 ± 12823.64 | 0.126 | 53209.56 ± 109015.13 | 6412.57 ± 5759.4 | 0.279 | 7072 ± 11403 | 6663 ± 9459 | 0.667 |

**Supplementary Table 2:** blood gas analysis results between the two groups. Values are shown as mean ± SD or median for non-parameters variables. P values are from t-test

|  | Total population | | | ICU patients | | | Internal medicine patients | | |
| --- | --- | --- | --- | --- | --- | --- | --- | --- | --- |
|  | CPE | Controls | p | CPE | Controls | p | CPE | Controls | p |
| pH | 7.48 ± 0.05 | 7.45 ± 0.09 | 0.055 | 7.49 ± 0.06 | 7.34 ± 0.30 | 0.484 | 7.47 ± 0.05 | 7.45 ± 0.08 | 0.090 |
| pO2 (mmHg) | 83.62 ± 35.13 | 77.33 ± 32.78 | 0.379 | 72.38 ± 24.76 | 62.00 ± 13.34 | 0.458 | 81.46 ± 24.33 | 72.73 ± 23.76 | **0.030** |
| pCO2 (mmHg | 35.49 ± 7.83 | 36.95 ± 11.09 | 0.517 | 34.00 ± 6.87 | 42.5 ± 27.23 | 0.406 | 36.70 ± 8.49 | 37.44 ± 11.23 | 0.782 |
| HCO_3_^-^ (mmol/L) | 26.10 ± 5.00 | 25.19 ± 5.12 | 0.433 | 21.10 ± 0.71 | 24.10 ± 4.82 | **0.050** | 26.47 ± 4.99 | 25.61 ± 4.82 | 0.371 |
| SO2 (%) | 92.60 ± 7.07 | 94.74 ± 5.17 | **0.048** | 91.57 ± 8.87 | 98.00 ± 1.73 | **0.053** | 97.24 ± 2.73 | 95.60 ± 3.07 | **0.020** |
| A-aDO2 (mmHg) | 199.02 ± 169.57 | 57.12 ± 72.53 | **0.0002** | 314.73 ± 116.94 | 190.64 ± 215.25 | **0.049** | 155.7 ± 157.3 | 52.97 ± 39.65 | **0.0002** |
| P/F | 230.28 ± 133.71 | 324.48 ± 124.19 | **0.001** | 153.62 ± 108.12 | 238.81 ± 134.93 | 0.261 | 267.0 ± 138.1 | 331.0 ± 127.5 | **0.008** |

Supplementary Table 3: Cox regression of intra-hospital mortality as univariate and multivariate analysis

|  | HR | 95% c.i. inferior limit | 95% c.i. superior limit | p-value |
| --- | --- | --- | --- | --- |
| Univariate |  |  |  |  |
| COVID | 0,54 | 0,11 | 2,62 | 0,45 |
|  |  |  |  |  |
| Multivariate |  |  |  |  |
| Age | 0,98 | 0,87 | 1,09 | 0,66 |
| COVID | 3,89x10^-8 | 0 | inf | 1 |
| Gender | 4,25x10^-9 | 0 | inf | 1 |
| Wells | 1,33 | 0,74 | 2,39 | 0,34 |
| Interventricular septum | 1,05 | 0,05 | 2,29 | 0,27 |
| Right Atrium | 1,1 | 0,67 | 1,78 | 0,72 |

Supplementary Table 4: Cox regression of 2-months mortality as univariate and multivariate analysis

|  | HR | 95% c.i. inferior limit | 95% c.i. superior limit | p-value |
| --- | --- | --- | --- | --- |
| Univariate |  |  |  |  |
| COVID | 0,45 | 0,16 | 1,22 | 0,12 |
|  |  |  |  |  |
| Multivariate |  |  |  |  |
| Age | 0,96 | 0,89 | 1,04 | 0,34 |
| COVID | 7,6x10^-9 | 0 | inf | 1 |
| Gender | 0,62 | 0,03 | 12,2 | 0,76 |
| Wells | 1,07 | 0,73 | 1,57 | 0,72 |
| Interventricular septum | 0,67 | 0,35 | 1,28 | 0,23 |
| Right Atrium | 1,09 | 0,89 | 1,34 | 0,41 |

**Supplementary Figure 1:** Correlation between platelet (PLT) counts and P/F and alveolar-artery oxygen difference in patients affected by COVID (panel A) and non-COVID (panel B) pulmonary embolism.
